# *De novo* transcriptome sequencing of *Isaria cateniannulata* and comparative analysis of gene expression in response to heat and cold stresses

**DOI:** 10.1371/journal.pone.0186040

**Published:** 2017-10-12

**Authors:** Dingfeng Wang, Liangde Li, Guangyuan Wu, Liette Vasseur, Guang Yang, Pengrong Huang

**Affiliations:** 1 Tea Research Institute, Fujian Academy of Agricultural Sciences, Fu’an, Fujian, China; 2 Key Laboratory of Integrated Pest Management for Fujian-Taiwan Crops, Ministry of Agriculture, Fuzhou, Fujian, China; 3 State Key Laboratory of Ecological Pest Control for Fujian and Taiwan Crops, Institute of Applied Ecology, Fujian Agriculture and Forestry University, Fuzhou, Fujian, China; 4 Department of Biological Sciences, Brock University, St Catharines, Ontario, Canada; 5 Key Laboratory of Green Control of Insect Pests (Fujian Agriculture and Forestry University), Fujian Province University, Fuzhou, Fujian, China; 6 Fujian-Taiwan Joint Center for Ecological Control of Crop Pests, Fujian Agriculture and Forestry University, Fuzhou, Fujian, China; Chinese Academy of Sciences, CHINA

## Abstract

*Isaria cateniannulata* is a very important and virulent entomopathogenic fungus that infects many insect pest species. Although *I*. *cateniannulata* is commonly exposed to extreme environmental temperature conditions, little is known about its molecular response mechanism to temperature stress. Here, we sequenced and *de novo* assembled the transcriptome of *I*. *cateniannulata* in response to high and low temperature stresses using Illumina RNA-Seq technology. Our assembly encompassed 17,514 unigenes (mean length = 1,197 bp), in which 11,445 unigenes (65.34%) showed significant similarities to known sequences in NCBI non-redundant protein sequences (Nr) database. Using digital gene expression analysis, 4,483 differentially expressed genes (DEGs) were identified after heat treatment, including 2,905 up-regulated genes and 1,578 down-regulated genes. Under cold stress, 1,927 DEGs were identified, including 1,245 up-regulated genes and 682 down-regulated genes. The expression patterns of 18 randomly selected candidate DEGs resulting from quantitative real-time PCR (qRT-PCR) were consistent with their transcriptome analysis results. Although DEGs were involved in many pathways, we focused on the genes that were involved in endocytosis: In heat stress, the pathway of clathrin-dependent endocytosis (CDE) was active; however at low temperature stresses, the pathway of clathrin-independent endocytosis (CIE) was active. Besides, four categories of DEGs acting as temperature sensors were observed, including cell-wall-major-components-metabolism-related (CWMCMR) genes, heat shock protein (Hsp) genes, intracellular-compatible-solutes-metabolism-related (ICSMR) genes and glutathione S-transferase (GST). These results enhance our understanding of the molecular mechanisms of *I*. *cateniannulata* in response to temperature stresses and provide a valuable resource for the future investigations.

## Introduction

Entomopathogenic hyphomycete fungi, such as *Beauveria bassiana* and *Metarhizium* spp., are very common and abundant in ecosystems and many are well known to control insects, nematodes, and plant pathogens [1, 2]. However, they are very susceptible to extreme temperatures (heat and cold) [3–5]. High temperatures affect the efficiency of entomopathogenic fungi as pest control agent by hindering fungal germination [4], growth [6, 7], and virulence [8]. The shelf life of hyphomycetes-based formulations can also be reduced by those factors [9]. Low temperature does not kill conidia of entomopathogenic fungi, but it stops or delays conidial germination [5], and facilitates storage of fungal formulations [3].

Entomopathogenic hyphomycetes have evolved complex molecular mechanisms to protect themselves from extreme environmental temperatures. Many stress-related genes and metabolic pathways have been identified in response to environmental stimuli. Since the fungal cell wall is the first line of defense against harsh environments, cell-wall-major-components-metabolism-related (CWMCMR) genes have been shown to play a critical role [10, 11]. The gene encoding β-1,3-glucan synthase (*MaFKS*) in *M*. *acridum* plays a role in the maintenance of cell wall integrity, hyperosmotic pressure tolerance, and conidiation [12]. Two GPI (glycosylphosphatidylinositol)-anchored protein Ecm33 orthologous genes, *Bbecm33* in *B*. *bassiana* and *Mrecm33* in *M*. *robertsii*, have been reported to be essential for cell wall integrity and multi-stress tolerance [13]. Both heat shock proteins (Hsps) and intracellular compatible solutes (e.g., trehalose, mannitol, glycerol) are important anti-stress agents [14, 15]. Under heat stress, in *M*. *anisopliae*, 10 Hsp genes and three orthologous trehalose-6-phosphate synthase genes are up-regulated [16]. The expression of *Hsp70* gene in *B*. *bassiana* is up-regulated by high (38°C) and low (4°C) temperatures, or UV stress [17]. Overexpression of the endogenous *Hsp25* gene in *M*. *robertsii* improves its tolerance to several stresses, including heat and cold [18]. Both Mas5 and Mdj1, the members of Hsp40 in *B*. *bassiana*, play a very important role in environmental adaptation and host infection [19, 20]. The deletion of mannitol-1-phosphate dehydrogenase (MPD) reduces the content of mannitol in *B*. *bassiana*, resulting in a decline of conidial tolerance to diverse stresses, including heat [21]. The ribosome pathway, endocytosis pathway and proteasome pathway are active in *M*. *anisopliae* under conditions of heat shock stress [16].

The exploration of stress-related genes provides opportunities for genetic improvement of fungal stress tolerance in order to develop high-efficiency and field-persistent mycoinsecticides and/or mycoacaricides. *Isaria cateniannulata* (Z. Q. Liang) Samson & Hywel-Jones [22] (formerly *Paecilomyces cateniannulatus* [23]) is a highly virulent entomopathogenic fungus belonging to hyphomycetes [23, 24]. It can infect insect pests such as Lepidoptera [23–25], Coleoptera [23], and Hemiptera [23, 26], as well as mites [27] and nematodes [28]. This fungus is one of the dominant entomopathogenic fungi in several ecosystems including pine plantation [29] and tea plantation [30]. However, *I*. *cateniannulata* genomic information and its molecular responses to temperature variation remain unknown.

Fortunately, *de novo* transcriptome assembly and digital gene expression (DGE) sequencing using the Illumina offer the most cost-effective choice for characterizing non-model organisms without a reference genome [31]. In this study, we present the first comprehensive transcriptome characterization of *I*. *cateniannulata* and explore the influence of heat and cold temperatures on gene expressions. Using the Illumina HiSeq2000, we generated over 29 million clean reads and these reads were assembled into 17,514 unigenes without a reference genome. DGE analysis was then employed to further validate the genes and metabolic pathways in response to high or low temperatures in *I*. *cateniannulata*. This first transcriptomic report of *I*. *cateniannulata* functional genes provides an essential database for developing strategies to enhance resilience of entomopathogenic hyphomycetes to temperature variation.

## Materials and methods

### Culture of *I*. *cateniannulata*

The wild-type strain ICBS918 of *I*. *cateniannulata* was used for this study. This strain was isolated from cadaver of *Homona coffearia* with a high virulence to *H*. *coffearia* and *Adoxophyes honmai* larvae [24]. This strain was cultured on potato dextrose agar (Difco) for 7 d at 26°C under the light/dark cycle of 14h/10h. After this period, three replicates of the culture were exposed separately to three temperatures: 4°C (cold or LT), 26°C (control or NT), and 39°C (heat or HT) for 4 h (for a total of nine replicates). Mycelia were then harvested for total RNA extraction.

### RNA isolation, library preparation, and sequencing

Total RNA was extracted using the SV total RNA isolation kit (Promega) according to the manufacturer’s protocol. The contamination and degradation of the extracted total RNA were preliminarily assessed on 1% agarose gels. The RNA integrity was checked with an Agilent Bioanalyzer 2100 system, and the purity and amount determined using an IMPLEN NanoPhotometer® spectrophotometer. Total RNA was finally quantified using the Qubit RNA Assay Kit in a Qubit 2.0 Flurometer according to the manufacture protocol (Life Technologies, CA, USA).

Equimolar quantities of the total RNA from the nine samples (1 μg from each sample) were combined into one pool as the input material for the transcriptome library, which acts as a reference library. To build DGE libraries for the nine samples (three replicates in each of the three treatments), 3 μg of extracted RNA were used. The cDNA library was constructed using the NEBNext Ultra RNA Library Prep Kit for Illumina (New England Biolabs, Ipswich, MA, USA) and the final cDNA library was selectively enriched by PCR and purified with the AMPure XP system (Beckman Coulter, Beverly, USA). The cDNA library was then sequenced on the Illumina HiSeq 2000 platform by the Novogene Bioinformatics Technology Co. Ltd (Beijing, China). Finally, 100-bp paired-end raw reads for transcriptome and 100-bp single-end raw reads for DGE were generated. All raw read sequences were deposited in the NCBI Short Read Archive (SRA) database under the accession number of SRP073968.

### *De novo* assembly and annotation

Prior to transcriptome assembly, raw sequencing reads were filtered through in-house Perl scripts to discard dirty reads, which included adaptors, unknown ‘N’ nucleotides (where the ‘N’ ratio was more than 10%), and low quality sequences (where the quality score was less than 5). Based on the clean reads, *de novo* transcriptome assembly into transcripts without a reference genome was carried out using short reads assembling program Trinity with min_kmer_cov set to 2 and all other parameters set by default [32]. For removing redundancy, if a component contained more than one assembled transcript, only the longest one was regarded as a unigene. To annotate the unigenes, we used NCBI BLAST 2.2.28+ with an E-value threshold of 10^−3^ in the database of eukaryotic orthologous groups (KOG), and with an E-value threshold of 10^−5^ in the NCBI non-redundant protein sequences (Nr) database, NCBI nucleotide collection (Nt) database and the Swiss-Prot protein database. Protein family (Pfam) was assigned using the HMMER 3.0 package. The database categories of Kyoto Encyclopedia of Genes and Genomes (KEGG) were assigned to the unigenes according to the KEGG Automatic Annotation Server (KAAS) online [33]. Based on BLASTX hits against the Nr and Swiss-Prot protein database (E value≤10^−5^), the gene ontology (GO) annotation of unigenes was obtained using Blast2GO v2.5 [34] and GO functional classification was performed using WEGO software [35]. The aligning results were compared against the Nr and Swiss-Prot databases with a priority order of Nr > Swiss-Prot to decide the unigenes’ direction and coding sequences (CDSs). When unigenes were unaligned to any of the above databases, ESTScan software [36] was used to predict the coding regions and sequence orientation.

### Analysis of DGE tags

Raw reads of DGE sequences were deposited in the NCBI Short Read Archive (SRA) database (http://www.ncbi.nlm.nih.gov/sra). Raw sequence data of the libraries for DGE profiling analyses were filtered to remove reads containing adaptors and reads with more than 10% unknown nucleotides, and reads with more than 50% of low-quality base (value≤5). Clean reads were mapped into the assembled transcriptome reference database and the read counts for each gene derived from the mapping results were achieved by RSEM [37]. All read counts were normalized using expected number of fragments per kilobase of transcript sequence per millions base pairs (FPKM), as described by Trapnell et al. [38]. The DESeq R package (1.10.1) was used for differential expression analysis of unigenes between the control (26°C) and the treatments (4°C or 39°C). To evaluate the reproducibility of DGE library sequencing, a Pearson correlation analysis was performed using the replicates between the various treatments. A corrected *P*-value of <0.05 (*P* values adjusted using the Benjamini & Hochberg method, *P*_adj_) was used as the threshold to judge the significant differences in gene expression. The heatmap of the differentially expressed genes (DEGs) was constructed using the R packages of ggplot2 and pheatmap.

In order to analyze the biological functions and metabolic pathways of the DEGs, DEGs were subject to GO functional analysis using Blast2GO v2.5 [34] and KEGG enrichment analysis using the KOBAS software [39]. Pathways with *P* value cutoff of ≤0.05 were considered significantly enriched by DEGs.

### Quantitative real-time PCR analysis

To confirm the DGE results, 18 genes were randomly selected for qRT-PCR verification. Specific primers for qRT-PCR were designed using the Primer Premier 5.0 software (Premier, Canada) and listed in the S1 Table. RNA for qRT-PCR analysis was extracted from the mycelia under the extreme temperature, as described above, using the SV total RNA isolation kit (Promega, USA). Reverse transcription was carried out according to the manufacturer’s protocol of GoScript™ Reverse Transcription System (Promega, USA). The qRT-PCR was run in the CFX96 Touch^TM^ Real-Time PCR Detection Systems (Bio-Rad, USA) using BRYT-Green-based PCR reaction. PCR amplification was performed in a total reaction mixture of 20 μL, containing 20 ng cDNA, 10 μL 2 × GoTaq® qPCR Master Mix (Promega, USA) and 0.2 μM of each primer. PCR was run with the standard thermal cycle conditions using the two-step qRT-PCR method: an initial denaturation at 95°C for 30s, followed by 40 cycles of 3 s at 95°C and 30 s at 60°C. Each reaction was run in triplicate, and the average threshold cycle (*C*_*t*_) was calculated for each replicate. The assembled glyceraldehyde-3-phosphate dehydrogenase unigene (GenBank accession no. KT944290) was employed as the internal gene and the relative expression of gene was calculated using the 2^-ΔΔCt^ method. The correlation of the fold change of the gene expression ratios between qRT-PCR and RNA-seq was checked by linear regression analysis in SPSS 17.0 software.

## Results

### Assembled transcriptome

As shown in Table 1, a total of 2.92 Gb nucleotides were obtained with a Q20 percentage of 95.5%. The percentage of unassigned base “N” was 5.95% and the average GC content, 55.17% (Table 1). By overlapping information in high-quality reads, 24,707 transcripts were generated, with an average length of 1,581 bp and an N50 of 2,664 bp. From these transcripts, 17,514 unigenes were obtained with a mean size of 1,197 bp (Table 2).

**Table 1:** Throughput and quality of RNA-seq of the reference library and the DGE libraries.

Library	Raw reads	Clean reads	Nucleotide (nt)	Q20 percentage (%)	N percentage (%)	GC percentage (%)
Reference library	33,741,894	29,230,890	2,923,089,000	95.50	5.95	55.17
NT1	10,935,341	10,568,185	1,056,818,500	95.00	0.00	55.12
NT2	7,873,444	7,653,904	765,390,400	95.00	0.00	55.07
NT3	7,655,454	7,435,760	743,576,000	95.00	0.00	56.30
LT1	9,569,387	9,351,945	935,194,500	96.00	0.01	55.47
LT2	10,030,572	9,782,547	978,254,700	96.00	0.01	55.01
LT3	11,786,747	11,521,026	1,152,102,600	96.00	0.01	55.17
HT1	9,328,176	9,094,778	909,477,800	96.00	0.01	55.61
HT2	14,377,463	14,052,602	1,405,260,200	96.00	0.01	55.22
HT3	11,799,489	11,524,052	1,152,405,200	96.00	0.01	56.11

Q20 percentage indicates the percentage of sequences with sequencing error rate lower than 1%. N percentage is the percentage of nucleotides which could not be sequenced.

**Table 2:** Summary of the *I*. *cateniannulata* transcriptome.

Category	Number				Total number	Mean length (bp)	N50 (bp)	Total nucleotide
	200–500 bp	500–1000 bp	1–2 kb	>2kb				
Transcript	7,633	4,250	5,718	7,106	24,707	1,581	2,664	39,071,015
Unigene	7,112	3,314	3,696	3,392	17,514	1,197	2,110	20,962,349

### Functional annotation and classification

**Fig 1:**
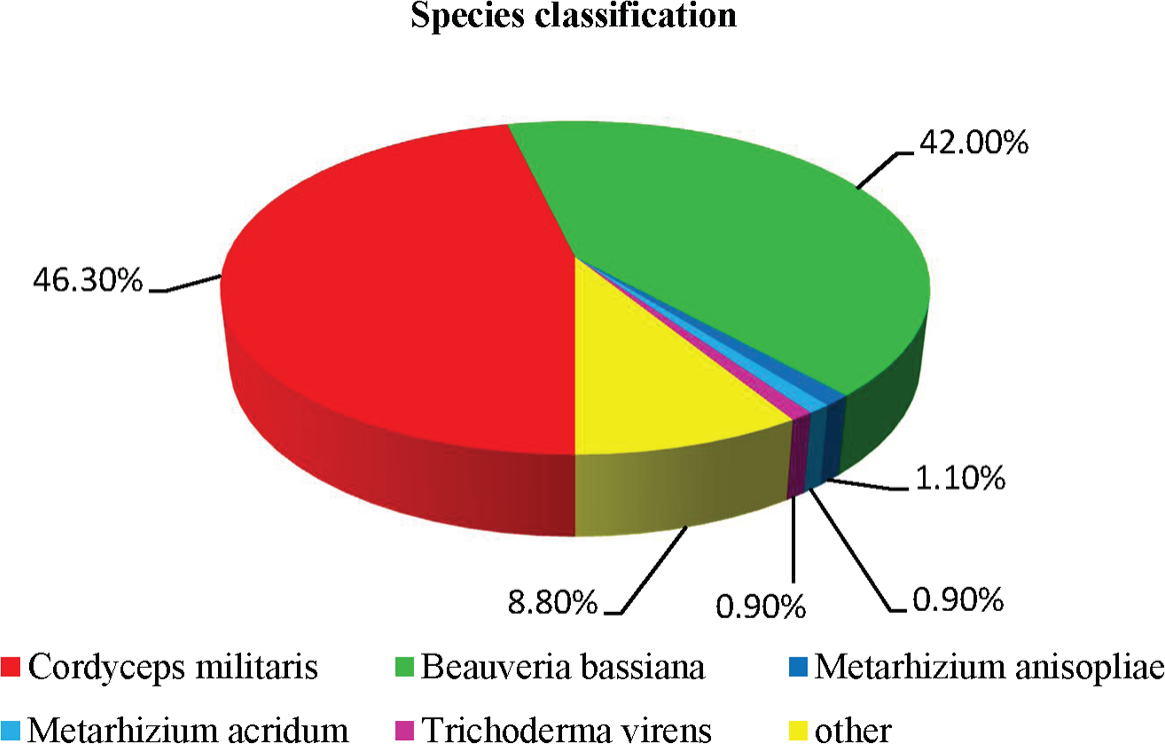
Species distribution of the first BLAST hits against the Nr database.

The 17,514 unigenes were accurately annotated by interrogating seven databases (Table 3): 65.34% (11,445) of unigenes could be annotated by BLASTx using the Nr database, 17.62% (3,087) by the Nt database, 16.58% (2,905) using the KEGG database, 37.41% (6,552) by the Swiss-Prot protein database, 45.22% (7,920) by the Pfam databases, 49.94% (8,748) according to the GO database, and 26.18% (4,586) according to the KOG database. In addition, 69.05% (12,095) of unigenes were annotated in at least one database, while only 8.81% (1,544) of unigenes were assigned to a homolog in all seven databases. A total of 17,204 coding sequences (12,174 predicted by BLASTX and 5,030 by ESTScan): 2,682 (15.59%) were smaller 200 bp, 12,939 (75.21%) were between 200 bp and 2,000 bp, and 1,583 (9.20%) were over 2,000 bp (S1 Fig). According to the BLASTX results of the Nr database, the sequence with the highest percentage of matching base (46.30%) was the *C*. *militaris* sequence, followed by the sequences of *B*. *bassiana* (42.00%) and *M*. *anisopliae* (1.10%) (Fig 1).

**Table 3:** Summary of the functional annotation of assembled unigenes.

Public database	No. of unigene hit	Percentage (%)
Annotated in NR	11,445	65.34
Annotated in NT	3,087	17.62
Annotated in KEGG	2,905	16.58
Annotated in SwissProt	6,552	37.41
Annotated in PFAM	7,920	45.22
Annotated in GO	8,748	49.94
Annotated in KOG	4,586	26.18
Annotated in all databases	1,544	8.81
Annotated in at least one database	12,095	69.05
Total unigene	17,514	100.00

**Fig 2:**
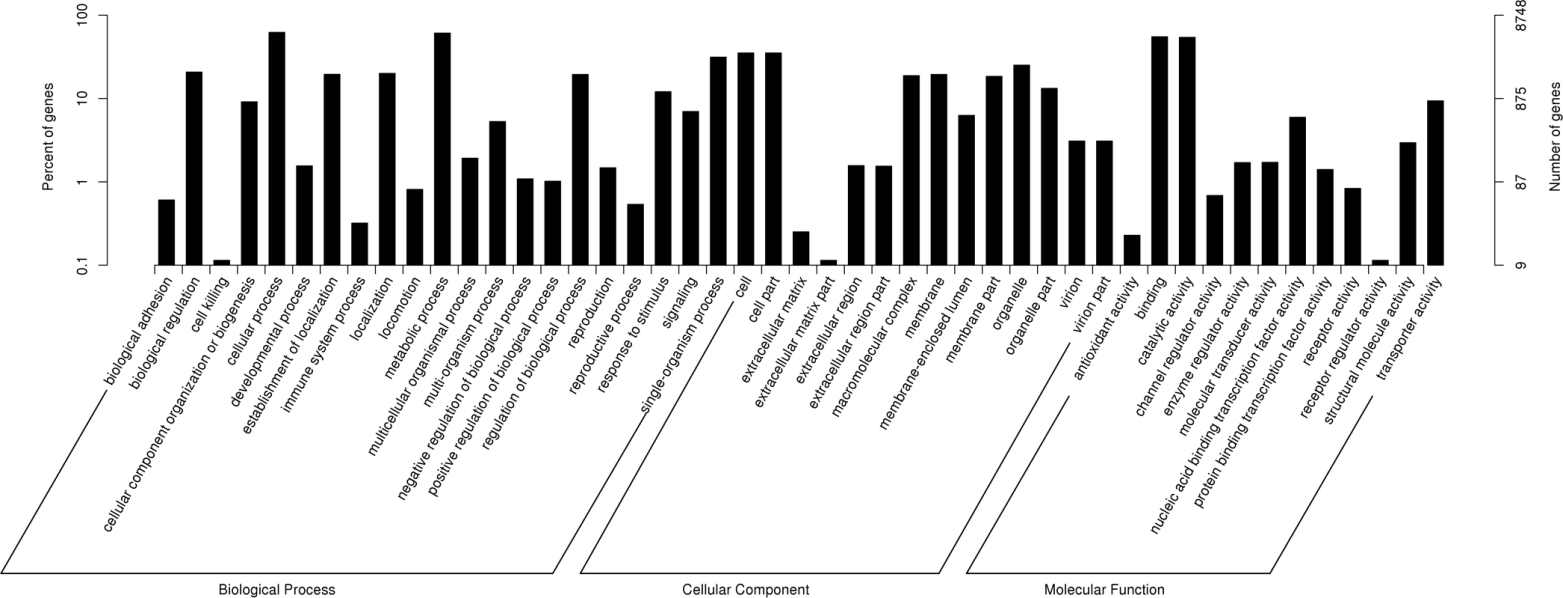
Histogram of gene ontology (GO) classification.

Gene ontology (GO) was used to classify the functions of the predicted *I*. *cateniannulata* unigenes. In total, 8,748 unigenes were classified into three different GO trees: biological process, cellular component, and molecular function (Table 3, Fig 2). The three different GO trees were divided into 47 functional groups, and further 1,179 functional terms (S2 Table). Seven groups were dominant clusters in GO classification: cellular process, metabolic process, single-organism process, cell, cell part, binding, and catalytic activity (Fig 2).

**Fig 3:**
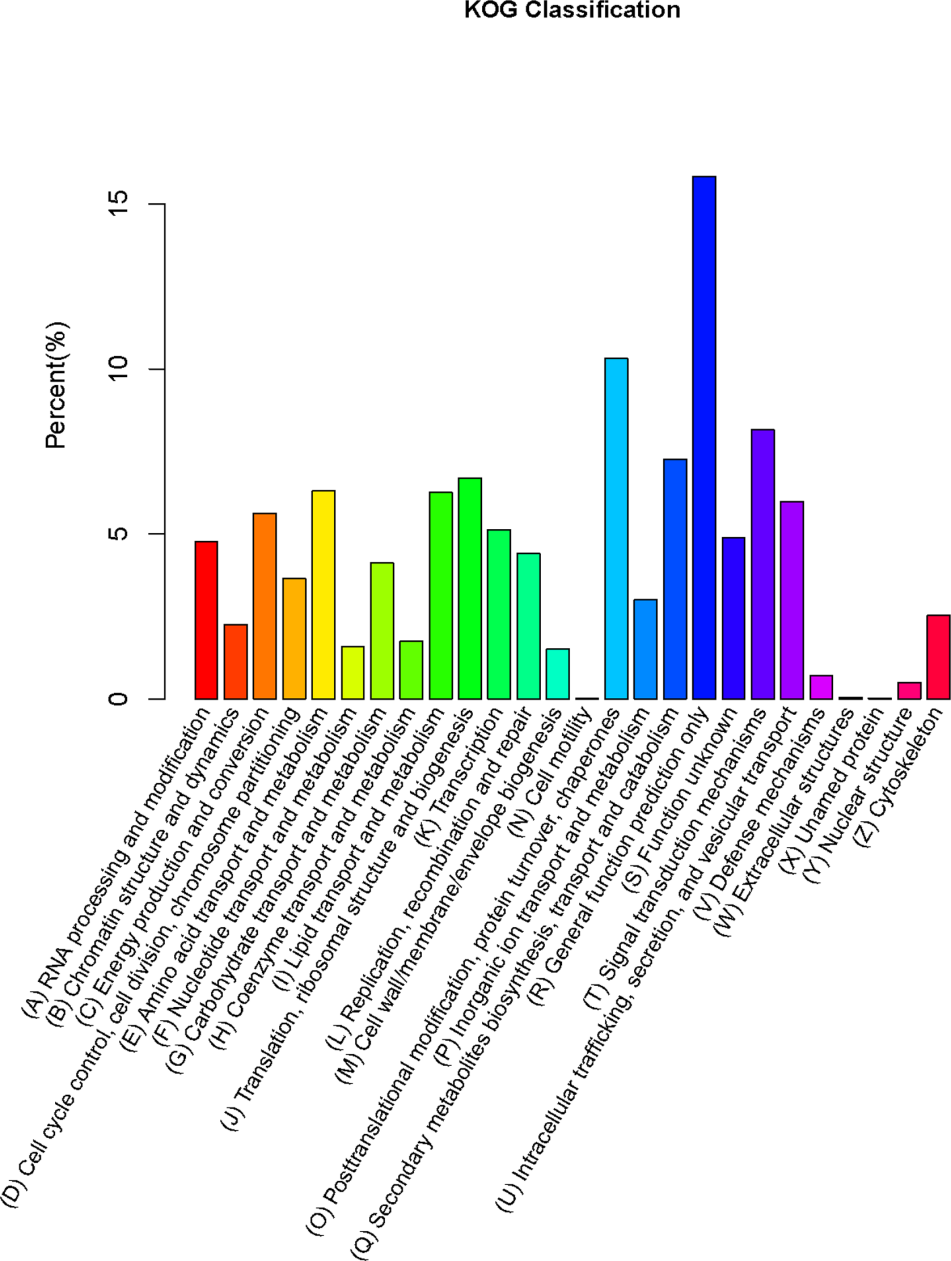
Histogram of KOG classification.

A total of 4,586 genes were assigned to KOG classification (Table 3, Fig 3) and grouped into 26 KOG categories (S3 Table). The largest category was “general function prediction only” (15.83%, 726), followed by “posttranslational modification, protein turnover, chaperones” (10.31%, 473), “signal transduction mechanisms” (8.16%, 374), “secondary metabolites biosynthesis, transport and catabolism” (7.26%, 333), and “translation, ribosomal structure and biogenesis” (6.69%, 307).

To further analyze the transcriptome of *I*. *cateniannulata*, the unigenes were searched against the KEGG pathway database. A total of 2,905 unigenes were annotated (Table 3) and assigned to 252 KEGG pathways. The most dominant pathways were “biosynthesis of amino acids” (ko01230, 115, 3.96%), “carbon metabolism” (ko01200, 95, 3.27%), and “ribosome” (ko03010, 91, 3.13%) (S4 Table).

### DGE library sequencing and mapping to the reference transcriptome

**Fig 4:**
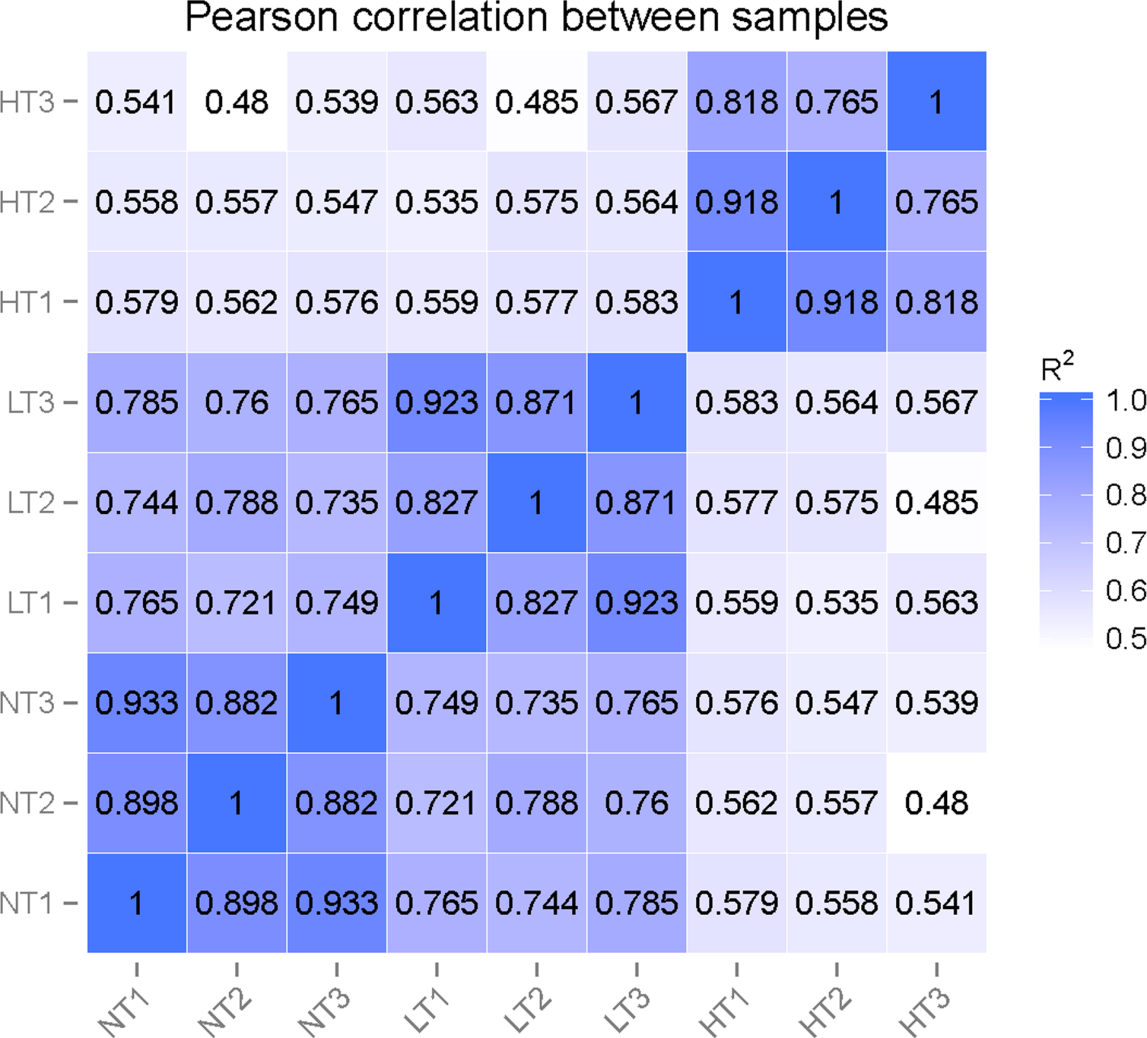
Correlation tests for the replicates. The abscissa represents the value log_10_ (FPKM + 1) of one duplicate; the ordinate represents the value log_10_ (FPKM + 1) of the other duplicate. R^2^ is the square of Pearson Correlation Coefficient.

Nucleotides of 0.74–1.41 Gb were obtained in each of the nine DGE libraries with a Q20 percentage of over 95%. The percentage of unassigned base “N” was below 0.01% and the average GC content was 55.01%-56.30% (Table 1). The clean reads of the nine DGE libraries were mapped to the above-constructed transcriptome reference database for each sample and the total mapped reads were above 96% for each replicate (S5 Table), which suggested the transcriptome was a reliable reference. The square of the Pearson correlation coefficient (R^2^) varied during 0.765–0.918 in HT treatment, 0.827–0.923 in LT treatment, and 0.882–0.933 in NT, indicating good operational stability and reliability of DEG library sequencing (Fig 4, S2 Fig).

### Differentially expressed genes and qRT-PCR verification

**Fig 5:**
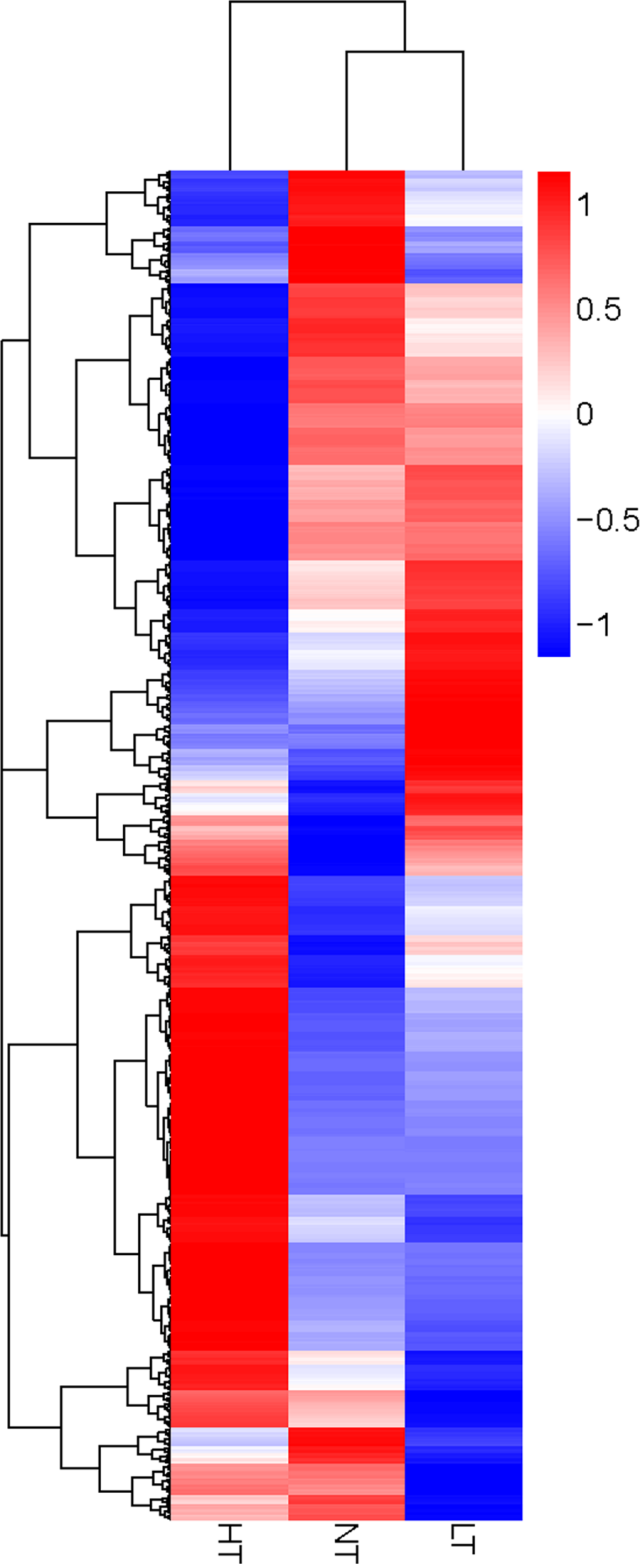
Heatmap of the expression profile of DEGs in the three temperature treatments. Each column represents a treatment, and each row represents a unigene. Differences in expression were shown in different colors. Data for gene expression level were normalized to z-score. Red represents up-regulated expression and blue represents down-regulated expression.

**Fig 6:**
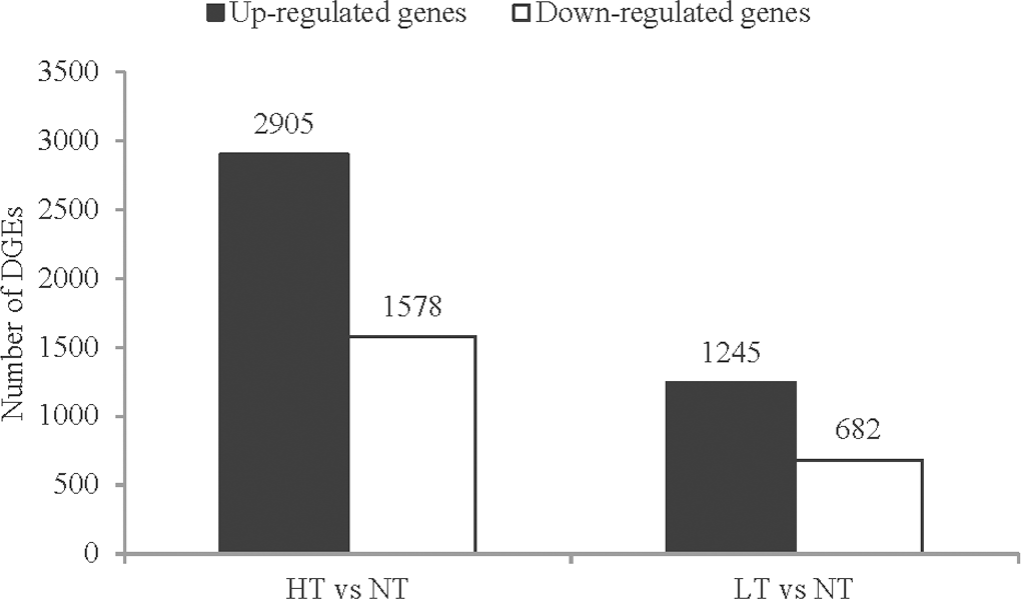
DEGs of *I*. *cateniannulata* when exposed to heat and cold treatments. The number of DEGs was obtained from comparisons in NT versus HT and NT versus LT.

**Fig 7:**
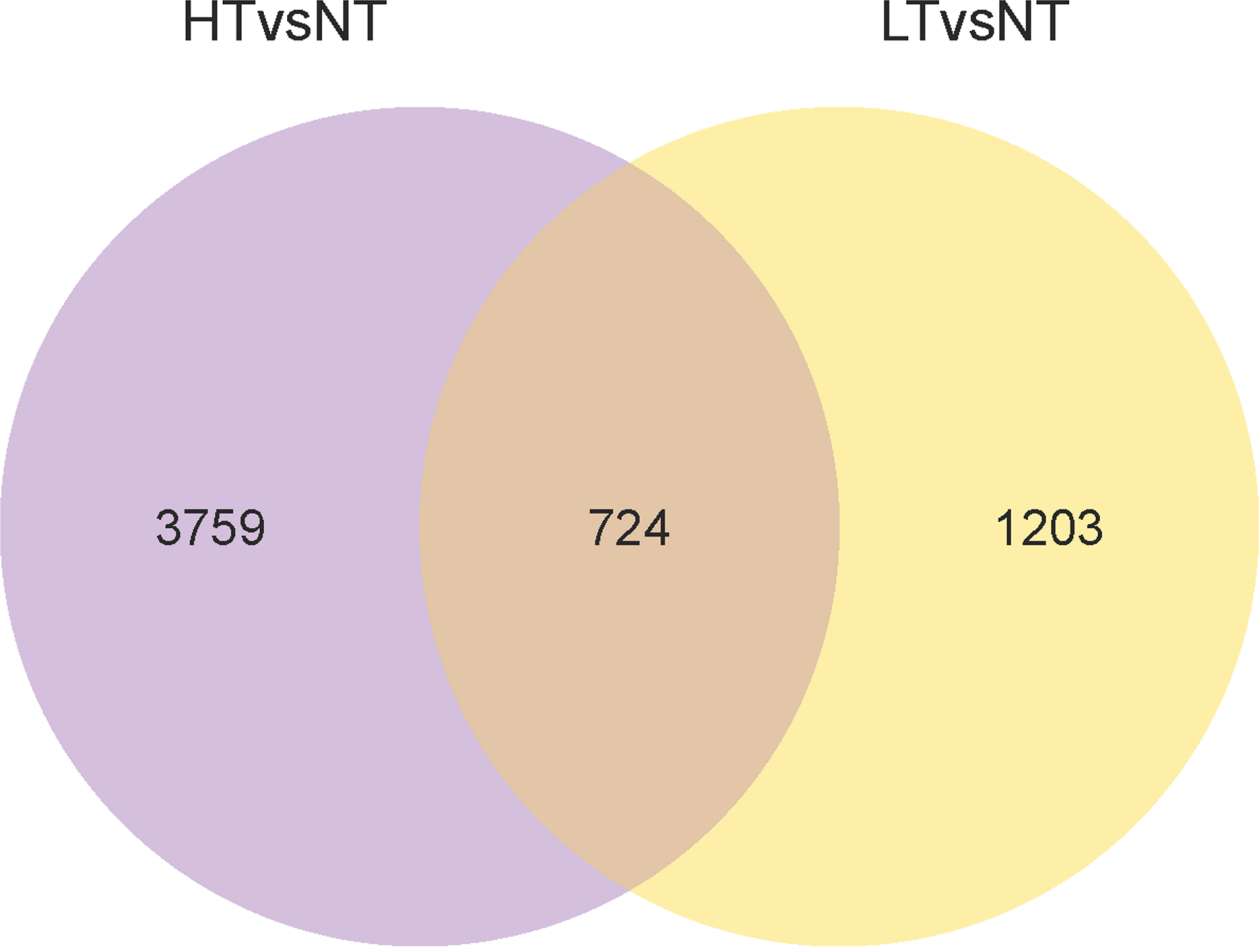
A Venn diagram describing the overlap of DEGs after treatments.

There were a total of 5,686 DEGs in response to extreme temperature stresses, and the expression profile in the heatmap (Fig 5) showed significant differences among the three temperature treatments. In HT treatment, the expression of 4,483 genes was significantly changed when compared with the control (NT), with 2,905 genes being up-regulated and 1,578 genes down-regulated. In LT treatment, the expression of 1,245 genes was up-regulated and 682 genes down-regulated when compared with the control (Fig 6, S6 Table). Overlapped transcripts between heat and cold stresses showed that there were 724 DEGs responding to both heat and cold stresses, 3,759 DEGs only responding to heat stress, and 1,203 DEGs only responding to LT treatment (Fig 7).

**Fig 8:**
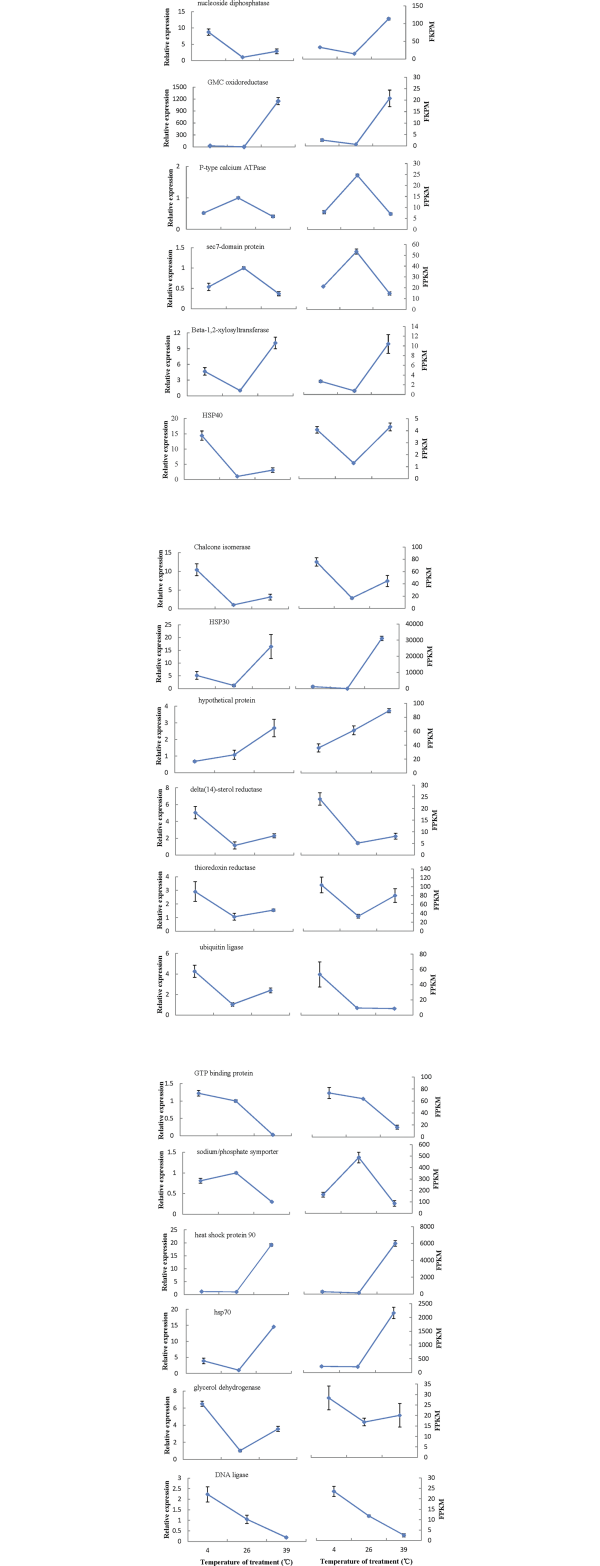
Expression profiles of eighteen unigenes uncovered by qRT-PCR (left side) and RNA-seq (right side). Both the qRT-PCR data and the FPKM value of RNA-seq are means of three biological replicates and bars represent SE.

**Fig 9:**
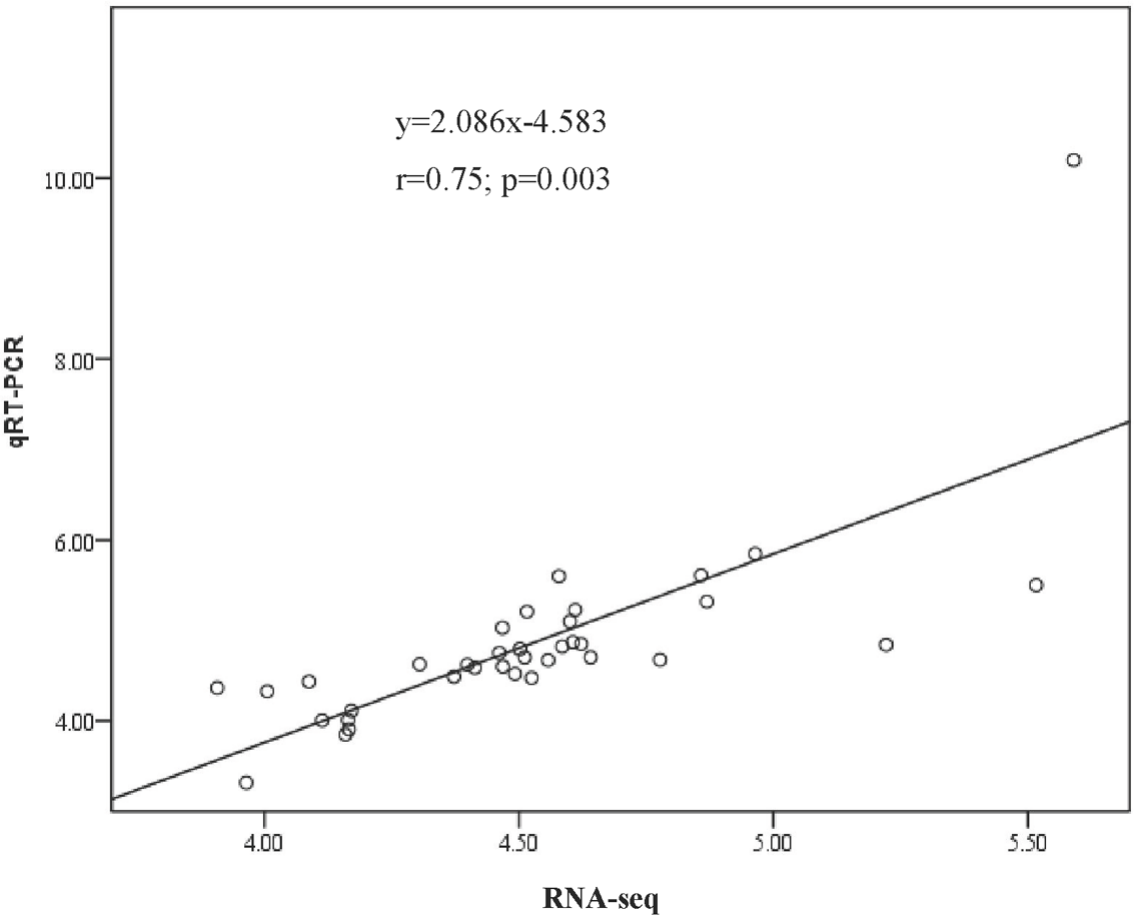
Correlation analysis of fold change data between qRT-PCR and RNA-seq. Data from qRT-PCR and RNA-seq are means of three replicates. Scatterplots were generated by the log2 expression ratios from RNA-seq (x-axis) and qRT-PCR (y-axis).

To verify the authenticity and reproducibility of the RNA-Seq results, 18 unigenes were randomly selected for qRT-PCR analysis and the expression profiles of the candidate unigenes by qRT-PCR were similar to the results of transcriptome analysis (Fig 8). The linear regression analysis showed significantly positive correlation of the relationship between gene expression ratios of qRT-PCR and RNA-seq was significantly positive (r = 0.75, *P* = 0.003) (Fig 9), confirming our transcriptomic data validity.

### DEGs in response to heat treatment

A total of 4,483 DEGs including 2,905 up-regulated and 1,578 genes down-regulated were identified in HT. GO functional classification of 4,483 DEGs showed that they could be categorized into 50 functional groups, belonging to three main GO domains: biological processes (20), cellular components (16), and molecular functions (14). Among these groups, we found that cellular process, metabolic process, and single-organism process in the biological process ontology, cell and cell part in the cellular component ontology, and binding and catalytic activity in the molecular function ontology were the most common annotation terms (S3 Fig).

**Fig 10:**
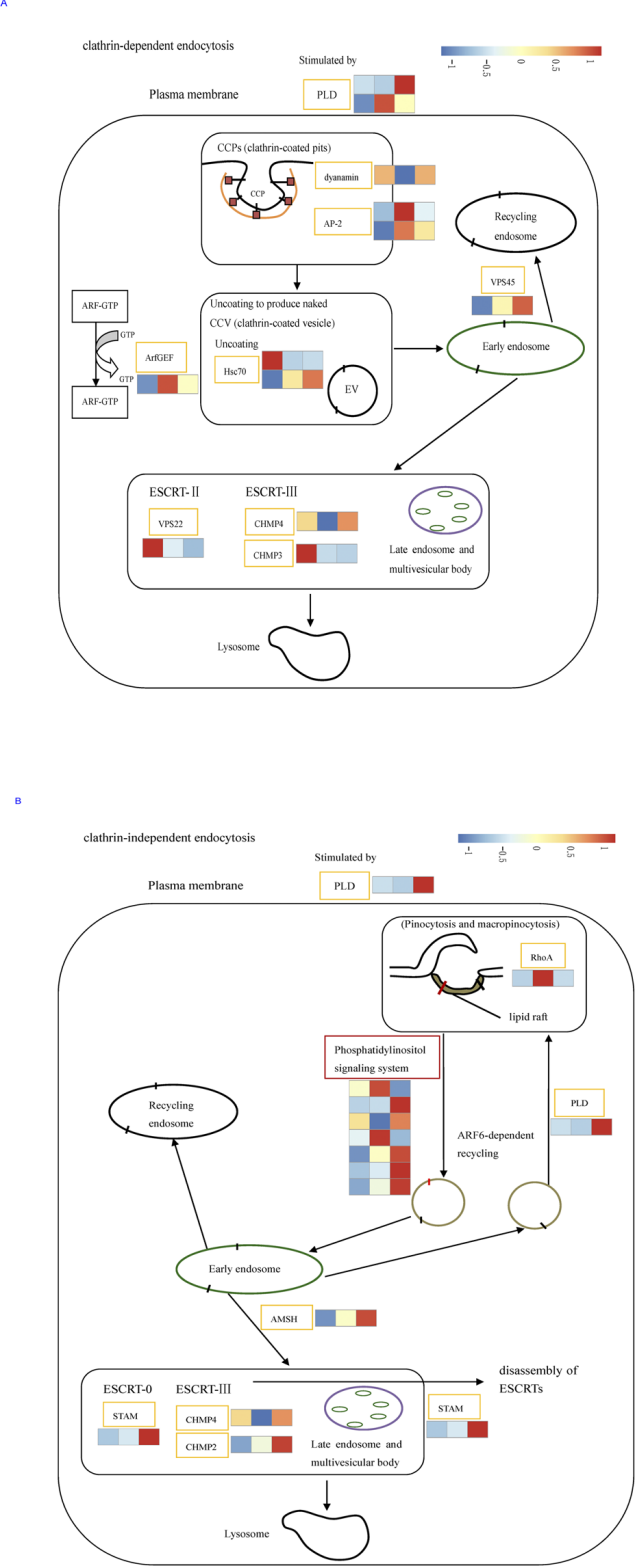
Differential expression genes involved in the pathway of endocytosis in *I*. *cateniannulata*. A. DEGs involved in clathrin-dependent endocytosis (CDE); B. DEGs involved in clathrin-independent endocytosis (CIE). Columns in each heatmap from left to right represented the value of FPKM: HT, NT and LT. And each row represents a gene. Expression differences are shown in different colors. Red means high expression and blue means low expression.

KEGG enrichment analysis showed that 8 pathways were significantly enriched (S7 Table). Among them, the ribosome biogenesis in eukaryotes was the most significantly enriched pathway, which contained 29 DEGs (28 down-regulated DEGs and 1 up-regulated DEG); the aminoacyl-tRNA biosynthesis was the second most significantly enriched pathway, which contained 18 DEGs (10 down-regulated DEGs and 8 up-regulated DEGs). However, we focused on the genes that were involved in endocytosis pathway, because of its ability to destroy misfolded and damaged proteins. After HT treatment, 12 DEGs related to clathrin-dependent endocytosis (CDE) were observed (Fig 10A). Among these 12 genes, 6 genes were up-regulated including PLD (comp7126_c0), dynamin (comp7305_c0), Hsc70 (comp7450_c0), VPS22 (comp2053_c0), CHMP4 (comp8162_c0) and CHMP3 (comp7520_c0); 6 genes were down-regulated including PLD (comp6558_c0), AP-2 (comp8686_c0 and comp6289_c0), Hsc70 (comp5575_c1), ArfGEF (comp4739_c0) and VPS45 (comp4862_c0).

In this study, four categories of DEGs functioning as temperature sensors were observed, including CWMCMR genes, Hsps, ICSMR genes and glutathione S-transferase (GST) genes. DEG analysis showed that there were 14 CWMCMR unigenes in the up-regulated unigenes, but no CWMCMR unigene was observed in the down-regulated unigenes (Table 4). In the Hsp superfamily, 25 unigenes from 6 Hsp families (Hsp100, Hsp90, Hsp70, Hsp60, Hsp40 and sHsp) were differentially regulated under heat treatments, including 24 up-regulated unigenes and 1 down-regulated unigene. Hsp40 family was the largest group with 8 family members and Hsp70 family was the second most abundant group with 6 family members (Table 5). Eight ICSMR genes including trehalose metabolism-related genes, mannitol metabolism-related genes, glycerol metabolism-related genes and arabinitol metabolism-related genes were differentially regulated (Table 6). Six GST genes were found to be up-regulated, and no GST gene was observed to be down-regulated (Table 7).

**Table 4:** Variation in the expression of CWMCMR genes when exposed to heat and cold treatments in *I*. *cateniannulata*.

Unigene ID	Annotation	HT/NT	LT/NT	Category
comp34522_c0	β-1,3-glucan synthase	☆	↑	Glucan metabolism-related genes
comp8138_c0	1,3-β-glucanosyltransferase	↑	↑	
comp2635_c0	exo-β-1,3-glucanase	↑	☆	
comp7516_c0	GPI-anchored cell wall β-1,3-endoglucanase	↑	↑	
comp4353_c0	endoglucanase EG-II	☆	↑	
comp3551_c0	endo-1,3(4)- β-glucanase	↑	☆	
comp49089_c0	endo-1,3(4)- β-glucanase 1	↑	↑	
comp48493_c0	glucan endo-1,4-β-glucanase activity	↑	↑	
comp4816_c0	cell wall glucanase	↑	☆	
comp4822_c0	xyloglucan endo-transglycosylase	↑	☆	
comp118120_c0	class 5 chitinase 1	↑	☆	Chitin/ chitosan metabolism-related genes
comp1058_c0	class III chitinase ChiA1	↑	☆	
comp129434_c0	chitinase II	↑	☆	
comp105600_c0	class V chitinase	↑	☆	
comp37786_c0	chitinase activity	☆	↑	
comp8305_c0	exochitinase	↑	↑	
comp1759_c0	cell wall mannoprotein CIS3	☆	↑	Mannoprotein metabolism-related genes
comp29349_c0	cell wall serine-threonine-rich galactomannoprotein Mp1	↑	☆	
comp7200_c0	mannan endo-1,6-alpha-mannosidase	☆	↑	
comp2210_c0	GPI anchored cell wall protein	☆	↑	GPI-anchored protein

“↑” indicates up-regulated-genes and “☆” indicates unigenes no differentially expressed.

**Table 5:** Variation of Hsp when exposed to heat and cold treatments in *I*. *cateniannulata*.

Unigene ID	Annotation	HT/NT	LT/NT	Category
comp7457_c0	heat shock protein Hsp98	↑	↑	Hsp100 family
comp7486_c0	heat shock protein 78	↑	☆	
comp5388_c0	heat shock protein 90	↑	☆	Hsp90 family
comp4106_c0	heat shock protein 70	↑	☆	Hsp70 family
comp4914_c0	hsp70-like protein	↑	☆	
comp7450_c0	heat shock protein 70	↑	☆	
comp6682_c0	heat shock protein 70	↑	☆	
comp5575_c1	heat shock protein 70	↓	☆	
comp6246_c0	chaperone protein dnaK	↑	☆	
comp7656_c0	heat shock protein 60	↑	☆	Hsp60 family
comp2707_c0	heat shock protein 60	↑	☆	
comp4697_c0	heat shock protein 60	↑	☆	
comp5545_c0	DnaJ domain containing protein	↑	☆	Hsp40/DnaJ family
comp8061_c0	DnaJ domain protein	↑	☆	
comp6354_c0	DnaJ domain protein	☆	↑	
comp2405_c1	Chaperone protein DnaJ	☆	↓	
comp7860_c0	DnaJ domain protein	☆	↑	
comp7085_c0	chaperone DnaJ	↑	☆	
comp6804_c0	DnaJ domain protein	↑	☆	
comp201_c0	DnaJ central domain	↑	☆	
comp7527_c0	DnaJ domain-containing protein	↑	☆	
comp2189_c0	DnaJ homolog subfamily A member 2	↑	☆	
comp27058_c0	DnaJ central domain	↑	↑	
comp2221_c0	30 kD heat shock protein	↑	↑	sHsp family
comp6572_c0	30 kD heat shock protein	↑	↑	
comp1380_c0	hsp20-like protein	↑	☆	
comp1717_c1	chaperonin 10 kD subunit	↑	☆	

“↑” indicates up-regulated-genes, “↓” indicates down-regulated-genes and “☆”indicates unigenes no differentially expressed.

**Table 6:** Variation in the expression of ICSMR genes when exposed to heat and cold treatments in *I*. *cateniannulata*.

Unigene ID	Annotation	HT/NT	LT/NT	Category
comp6260_c0	trehalase precursor	☆	↑	Trehalose metabolism-related genes
comp55474_c0	trehalose-phosphatase	↑	↑	
comp1429_c0	trehalose synthase	☆	↑	
comp2294_c0	trehalose-6-phosphate synthase (tps1)	↓	☆	
comp7547_c0	mannitol-1-phosphate dehydrogenase	↑	☆	Mannitol metabolism-related genes
comp13549_c0	mannitol-1-phosphate dehydrogenase	↑	☆	
comp2518_c0	mannitol dehydrogenase	↓	☆	
comp6842_c1	glycerol dehydrogenase Gcy1	↑	↑	Glycerol metabolism-related genes
comp6167_c0	glycerophosphoryl diester phosphodiesterase	☆	↑	
comp10601_c0	glycerophosphoryl diester phosphodiesterase	☆	↑	
comp7775_c0	triacylglycerol lipase	↑	↑	
comp5307_c0	D-arabinitol 2-dehydrogenase	↑	☆	Arabinitol metabolism-related genes

“↑” indicates up-regulated-genes, “↓” indicates down-regulated-genes and “☆”indicates unigenes no differentially expressed.

**Table 7:** Variation in the expression of GSTs when exposed to heat and cold treatments in *I*. *cateniannulata*.

Unigene ID	Annotation	HT/NT	LT/NT	Category
comp72432_c0	glutathione S-transferase kappa 1	↑	☆	glutathione S-transferase
comp5315_c0	glutathione S-transferase	↑	☆	
comp3427_c0	glutathione S-transferase	↑	☆	
comp7962_c0	glutathione S-transferase domain-containing protein	↑	☆	
comp9890_c0	glutathione S-transferase	↑	☆	
comp7963_c0	glutathione S-transferase domain-containing protein	↑	↑	

“↑” indicates up-regulated-genes and “☆”indicates unigenes no differentially expressed.

### DEGs in response to cold treatment

In comparison with NT, 1,927 DEGs were found in LT including 1,245 up-regulated genes and 682 down-regulated genes. All DEGs were categorized into 49 functional groups by GO functional classification, including biological processes (20), cellular components (14), and molecular functions (15). Among these groups, we found cellular process, metabolic process, and single-organism process in the biological process ontology, cell, cell part, and membrane in the cellular component ontology, and binding and catalytic activity in the molecular function ontology were the dominant clusters (S4 Fig).

The DEGs were highly enriched in polycyclic aromatic hydrocarbon degradation, phosphatidylinositol signaling system, tyrosine metabolism, inositol phosphate metabolism and drug metabolism—other enzymes by KEGG analysis (S7 Table). We also focused on the genes that were related to endocytosis pathway. After LT treatment, 10 DEGs related to clathrin-independent endocytosis (CIE) were up-regulated including PLD (comp7126_c0), PTEN (comp5571_c0),PLC (comp7099_c2), E3.1.3.56 (comp7092_c0 and comp4008_c0), PKC (comp5597_c0), AMSH (comp3635_c0), STAM(comp7300_c0), CHMP4 (comp8162_c0), CHMP2 (comp4090_c0), PTEN (comp5571_c0), PLC (comp7099_c2), E3.1.3.56 (comp7092_c0 and comp4008_c0) and PKC (comp5597_c0), while 3 of them were down-regulated including RhoA (comp6998_c0), PI4K(comp5407_c0) and PLC (comp7040_c0) (Fig 10B).

DEG analysis revealed that only CWMCMR (11) (Table 4), ICSMR (7) (Table 6), and GST unigenes (1) (Table 7) were up-regulated, and Hsp unigene (7) were differentially regulated including 6 members up-regulation and 1 member down-regulation (Table 5). In addition, one eubacteria-like cold shock protein (CSP) homologue (comp1755_c0) and one glycine-rich RNA binding protein (GRP) homologue (comp7081_c0) were found to be up-regulated under cold stress.

### DEGs in response to both heat and cold treatments

A total of 724 co-regulated DEGs (Fig 7) including 496 up-regulated and 228 down-regulated genes were identified in both heat and cold treatment. All DEGs were categorized into 45 functional groups by GO functional classification, including biological processes (21), cellular components (15), and molecular functions (9). Among these groups, we found cellular process, metabolic process, and single-organism process in the biological process ontology, cell, cell part, and organelle in the cellular component ontology, and binding and catalytic activity in the molecular function ontology were the dominant clusters (S5 Fig).

The co-regulated DEGs were highly enriched in glycolysis / gluconeogenesis, regulation of mitophagy–yeast, sulfur metabolism, glycerolipid metabolism and ABC transporters by KEGG analysis (S7 Table). Besides, there were two co-regulated DEGs, PLD (comp7126_c0) and CHMP4 (comp8162_c0), related to endocytosis pathway. DEG analysis revealed that 5 CWMCMR genes (Table 4), 4 Hsp genes (Table 5), 3 ICSMR genes (Table 6) and 1GST gene (Table 7) were both up-regulated under heat and cold treatments.

## Discussion

As the genomic information of *I*. *cateniannulata* was unavailable, and the molecular response mechanisms of the species to environmental variation not well understood, we used next-generation sequencing technology and transcriptome analysis as an alternative for in-depth analysis of molecular responses of *I*. *cateniannulata* to high and cold temperatures. In the present study, the transcriptome characterization of *I*. *cateniannulata* generated 29.2 million clean reads and assembled into 17,514 unigenes with a mean size of 1,197 bp. While 12,095 (69%) unigenes were successfully annotated using the public databases, 5,419 (31%) unigenes had no homologous sequences to public databases. This indicates that *I*. *cateniannulata* may contain several species-specific unigenes. The variation in number of unigenes and the expression profiles of DEGs under heat and cold temperature stresses suggests that the molecular responses of *I*. *cateniannulata* to high and low temperatures may greatly vary as reported in *Pyropia yezoensis* [40]. Though the cold temperature slows down the metabolic rates and results in the dormancy in organism, we still identified many DEGs in metabolic pathways under cold temperature treatment in our study. It maybe the cold temperature treatment (4°C for 4h) was not devastating to *I*. *cateniannulata*, and there were many DEGs have been activated. Our result is well in accordance with the study of *Pyropia yezoensis* in response to chilling and freezing stress [40], *Chrysanthemum nankingense* under low temperature [41], and *Anthurium andraeanum* under cold stress [42].

Endocytosis involves cells take up molecules (such as proteins) via vesicles, which is essential for cell-to-cell communication and cellular responses to external stimuli in all eukaryotic cells [43, 44]. Endocytosis pathway is significantly enriched in lotus (*Nelumbo Adans*) [45] and filamentous fungus (*M*. *anisopliae*) [16] under conditions of heat shock stress. And this pathway is also induced by cold stress in amur carp (*Cyprinus carpio haematopterus*) [46]. Endocytosis pathway contains two major categories: CDE and CIE [47]. CDE is well characterized and highly conserved cellular process from humans to fungi [48]. After heat treatment, the pathway of CDE was activated in *I*. *cateniannulata* as reported in *M*. *anisopliae* [16]. Dynamin is one of critical factors for different stages of CDE [49]. It was significantly up-regulated in *I*. *cateniannulata* under heat stress. Here, one *Hsc70* gene (comp7450_c0) up-regulation and the down-regulation of comp5575_c1 were observed after heat stress. Hsc70 play a very important role in the ATP-dependent dissociation of clathrin from clathrin coated vesicles (CCVs) and other key processes in CDE [50]. Late endosomes (also known as multivesicular bodies, MVBs) is one of the major trafficking hub of the endocytic pathway, which formation is essential for cells to destroy misfolded and damaged proteins [47]. The endosomal sorting complex required for transport (ESCRT) plays a very important role in the MVB sorting pathway [47]. In this study, three genes involved in the ESCRT pathway, including ESCRT-Ⅱ (VPS22) and ESCRT-Ⅲ (CHMP3 and CHMP4) were up-regulated under heat stress. It was speculated that the activation of CDE would facilitate the elimination of misfolded and damaged proteins, which caused by heat shock in *I*. *cateniannulata*. Apart from CDE, CIE pathway have been found in eukaryotic cells, which mediates many biological processes [51]. In this study, CIE was activated when *I*. *cateniannulata* was exposed to cold treatment. The gene encoding AMSH (associated molecule with a Src homology 3 domain), a deubiquitinating enzyme, was up-regulated as well as, three genes involved in the ESCRT pathway, ESCRT-0 (STAM (signal transducing adaptor molecule)) and ESCRT-Ⅲ (CHMP (charged multivesicular body proteins) 2 and 4). These results suggest that the activation of CIE pathway would accelerate the removal of denatured proteins caused by low temperature stress in *I*. *cateniannulata*.

A total of 20 CWMCMR genes were up-regulated in response to high and/or low temperatures. Cell wall plays a very important role in protecting fungi against a variety of harsh environments such as heat, cold, desiccation and osmotic stress [52]. In this study, 1 β-1,3-glucan synthase orthologous gene (comp34522_c0) was up-regulated under cold temperature. We suggest that the cell may synthesize more β-1,3-glucan to enhance cell wall tensile strength against cold temperature. β-1,3-glucan has a coiled spring-like structure that confers a degree of elasticity and tensile strength to the cell wall [11]. β-1,3-glucan synthase (MaFKS)-RNAi transformants of entomopathogenic fungus *M*. *acridum* are more sensitive to agents that disturb the cell wall or cell membrane and to hyperosmotic stress in comparison with the wild type [12].

GPI-anchored protein orthologous gene (comp2210_c0) was up-regulated in response to cold temperature. GPI-anchored protein may not only play a role in maintaining the fungal cell wall integrity [13, 53], but also contribute to their multi-stress tolerance [13]. So the up-regulation of orthologous genes (comp2210_c0) of GPI-anchored protein may enhance the cell wall integrity and offer more resistance to cold temperature. Other up-regulated genes such as glucan-metabolism-related genes, chitin/chitosan-metabolism-related genes, and mannoprotein-metabolism-related genes, might have also been involved in maintaining cell wall integrity and increasing tensile strength against heat and cold. However, the exact functions of these genes are elusive.

Twenty-seven Hsp genes from 6 major families (Hsp100, Hsp90, Hsp70, Hsp60, Hsp40 and sHsp) were differentially regulated when exposed to heat and/or cold in *I*. *cateniannulata*. Hsps are ubiquitous in all prokaryotes and eukaryotes and can be induced by several kinds of stresses, including extremes temperatures, desiccation, and toxic substances [54–56]. In the filamentous fungus *M*. *anisopliae*, 10 Hsp genes are up-regulated in heat-treated conidia [16]. Here, one *hsp98* homolog (comp7457_c0) was up-regulated in response to both heat and cold and one *hsp78* homolog (comp7486_c0) was up-regulated in response to cold treatment. Hsp100 family is a major heat-regulated protein family in several species [57]. The *Neurospora crassa* Hsp98 protein, a member of hsp100 family, is highly expressed in response to heat shock [58]. Hsp78, a member of the Clp/Hsp100 family in *S*. *cerevisiae*, is required for the maintenance of mitochondrial function under heat stress [59]. *Hsp90* homolog (comp5388_c0) was up-regulated upon exposure to heat. All eukaryotic cells produce prominent heat-shock protein with the molecular weight ranging from 80 to 90 kD, which are classified as the Hsp90 family [14, 60]. *Hsp90* transcription is significantly induced when exposed to various abiotic stresses such as heat, cold, and oxygen deprivation [61, 62].

Interestingly, 5 members of Hsp70 family in *I*. *cateniannulata* were up-regulated in response to heat and might be invovled with the removal of denatured proteins. This is in agreement with other studies. For example, Hsp70 can prevent the aggregation of unfolding proteins and even refold aggregated proteins under heat [63]. The fungus *B*. *emersonii* has 10 putative Hsp70 homologs, and all Hsp70 genes (except for *Hsp70-4* and *Hsp70-6*) are induced to different degrees upon exposure to heat [64]. The yeast *S*. *cerevisiae* has 14 Hsp70 genes, in which cytosolic Hsp70-Ssa (Ssa1, Ssa2, Ssa3 and Ssa4) is heat-inducible [65]. The transcriptional level of *Hsp70* is up-regulated in *B*. *bassiana* when exposed to heat (38°C), cold (4°C), or UV stress [17].

Three *Hsp60* homologs were up-regulated under heat in *I*. *cateniannulata*. The *Hsp60* gene expression levels are also up-regulated in the *Aspergillus fumigatus*, *A*. *terreus* and *Scedosporium apiospermum* under heat [66]. In addition, 10 *Hsp40/DnaJ* homologs were up-regulated under heat and/or cold temperatures. Hsp40 family, also known as J-protein family, is the largest class of Hsp70 cofactors, which bind nonnative proteins and deliver them to Hsp70 [67, 68]. Both Mas5 and Mdj1, the members of Hsp40 in *B*. *bassiana*, have been shown to be indispensable for the environmental adaptation and virulence [19, 20].

In our study, 2 *Hsp30* homologs (comp2221_c0 and comp6572_c0) were up-regulated under both heat and cold temperatures, and 1 *Hsp20* (comp1380_c0) and 1 *Hsp10* homologs (comp1717_c1) up-regulated when exposed to heat. The expression of *Hsp23*, a small heat-shock protein gene in *Trichoderma virens* is increased when the fungus is grown at extreme temperatures (4, 10 or 41°C) [69]. A small heat shock protein gene *hsp25* is up-regulated in *M*. *robertsii* in response to extreme temperatures (4, 35, and 42°C), and overexpression of *hsp25* improves the growth of *M*. *robertsii* when exposed to heat [18]. The sHsps of *A*. *nidulans* have been shown to take part in resisting adverse conditions, including heat and cold as well as oxidative/osmotic stresses [70].

Three trehalose-metabolism-related genes were up-regulated after heat and/or cold treatments in *I*. *cateniannulata*. Trehalose is present in a wide range of organisms, and could serve as a stabilizer and protectant of proteins and cellular membranes against a variety of stresses such as heat, cold, oxidation, and desiccation [71]. Under heat or chemical stress, the increasing of trehalose in the cell, which associated with up-regulation of the trehalose-6-P phosphatase transcript in arbuscular mycorrhizal (AM) fungi *Glomus intraradices* was observed [72]. Several trehalose accumulation-related genes are up-regulated in *M*. *anisopliae* in response to heat [16].

Here, two mannitol 1-phosphate dehydrogenase (MPD) orthologous genes (comp7547_c0 and comp13549_c0) showed up-regulation in response to heat. It was speculated that the up-regulation of MPD would increase the content of mannitol in *I*. *cateniannulata*. One D-arabinitol 2-dehydrogenase orthologous gene (comp5307_c0) was up-regulated under heat treatment. D-arabitol is one of the polyols found most frequently in fungi [73], which may act as adversity protectant [74]. In our study, 2 glycerol-metabolism-related genes were up-regulated when exposed to cold treatment, and 2 glycerol-metabolism-related genes were up-regulated in response to both heat and cold treatment in *I*. *cateniannulata*. The up-regulation of glycerol-metabolism-related genes may contribute to the increasing glycerol content against heat and cold temperatures.

GSTs play a very important role in response to oxidative stress by removing reactive oxygen species and regenerate S-thiolated proteins [75]. The GSTs involved in protecting *Schizosaccharomyces pombe* cells from damage causing by oxidative stress [76]. In our study, 6 GST genes were up-regulated under heat treatment, and 1 GST gene was up-regulated under cold treatment. We suggest that GST genes up-regulation might protect *I*. *cateniannulata* cells against damage resulting from oxidative stress induced by heat and cold treatments.

When *I*. *cateniannulata* was exposed to cold stress, the homologue of CSP (comp1755_c0) and GRP (comp7081_c0) were up-regulated. This is well in accordance with *M*. *anisopliae*’s CSP (CRP1) and GRP (CRP2) homologue, which play a key role in against cold stress [77].

## Conclusions

The combination of RNA-seq and DGE analysis based on next generation sequencing technology provided comprehensive information on gene expression of *I*. *cateniannulata*, an entomopathogenic fungus for which little genomic information was available. Many DEGs of *I*. *cateniannulata* were identified under heat and cold temperatures with significant differences in molecular responses. In this study, we mainly focused on endocytosis pathway and identified several genes that were either up or down regulated when exposed to changing temperatures. Candidate stress-related genes may be useful tools for improvement of strain tolerance against extreme environmental temperatures in *I*. *cateniannulata*.

## Supporting information

S1 Fig Length distribution of the coding sequences. (TIF)

S2 Fig Correlation tests for the replicates. (TIF)

S3 Fig GO classification of DEGs under HT. (TIF)

S4 Fig GO classification of DEGs under LT. (TIF)

S5 Fig GO classification of DEGs under both HT and LT. (TIF)

S1 Table Primers for qRT-PCR. (XLSX)

S2 Table GO classifications of assembled unigenes. (XLS)

S3 Table KOG_classification of assembled unigenes. (XLS)

S4 Table KEGG_classification of assembled unigenes. (XLS)

S5 Table sequence alignment between reads and transcriptome. (XLSX)

S6 Table DEGs under heat and cold stresses. (XLSX)

S7 Table KEGG enrichment analysis of DEGs. (XLSX)
