# Supplementary figures and images for: De novo transcriptome sequencing of Isaria cateniannulata and comparative analysis of gene expression in response to heat and cold stresses
Source: PLoS One. 2017 Oct 12;12(10):e0186040. doi: 10.1371/journal.pone.0186040 (PMC5638334; doi:10.1371/journal.pone.0186040)

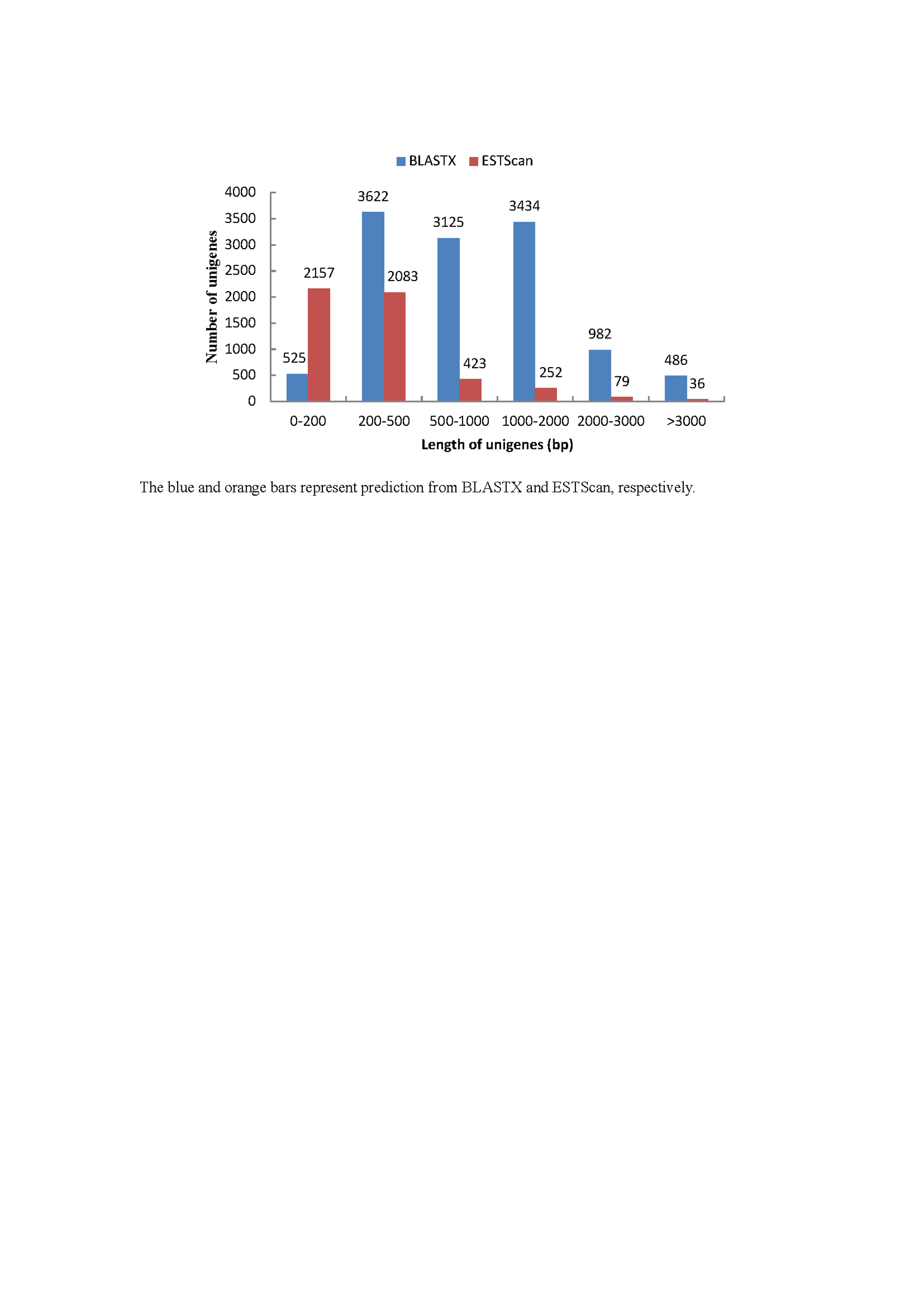

Supplement: S1 Fig — (TIF) [file pone.0186040.s001.tif]

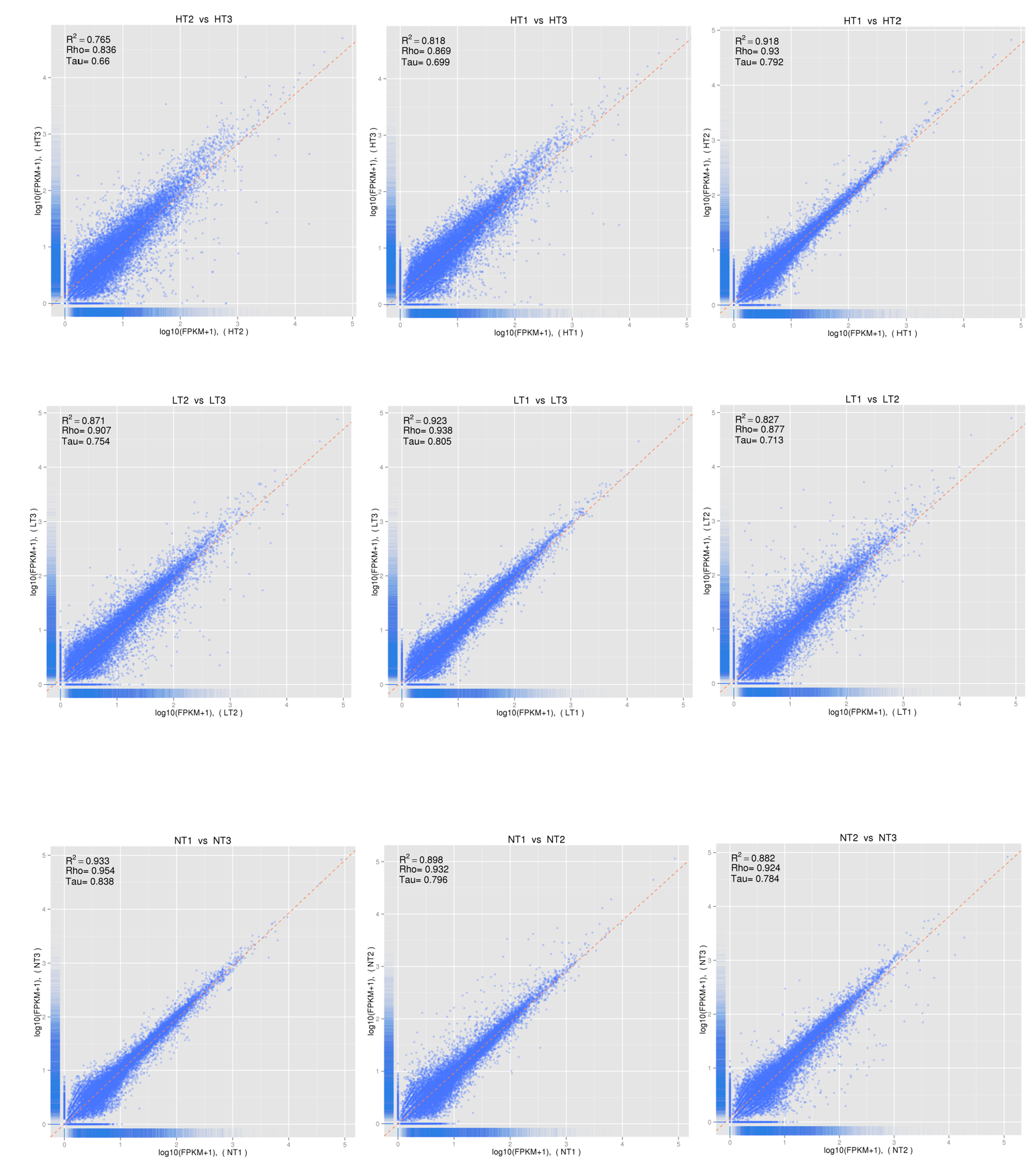

Supplement: S2 Fig — (TIF) [file pone.0186040.s002.tif]

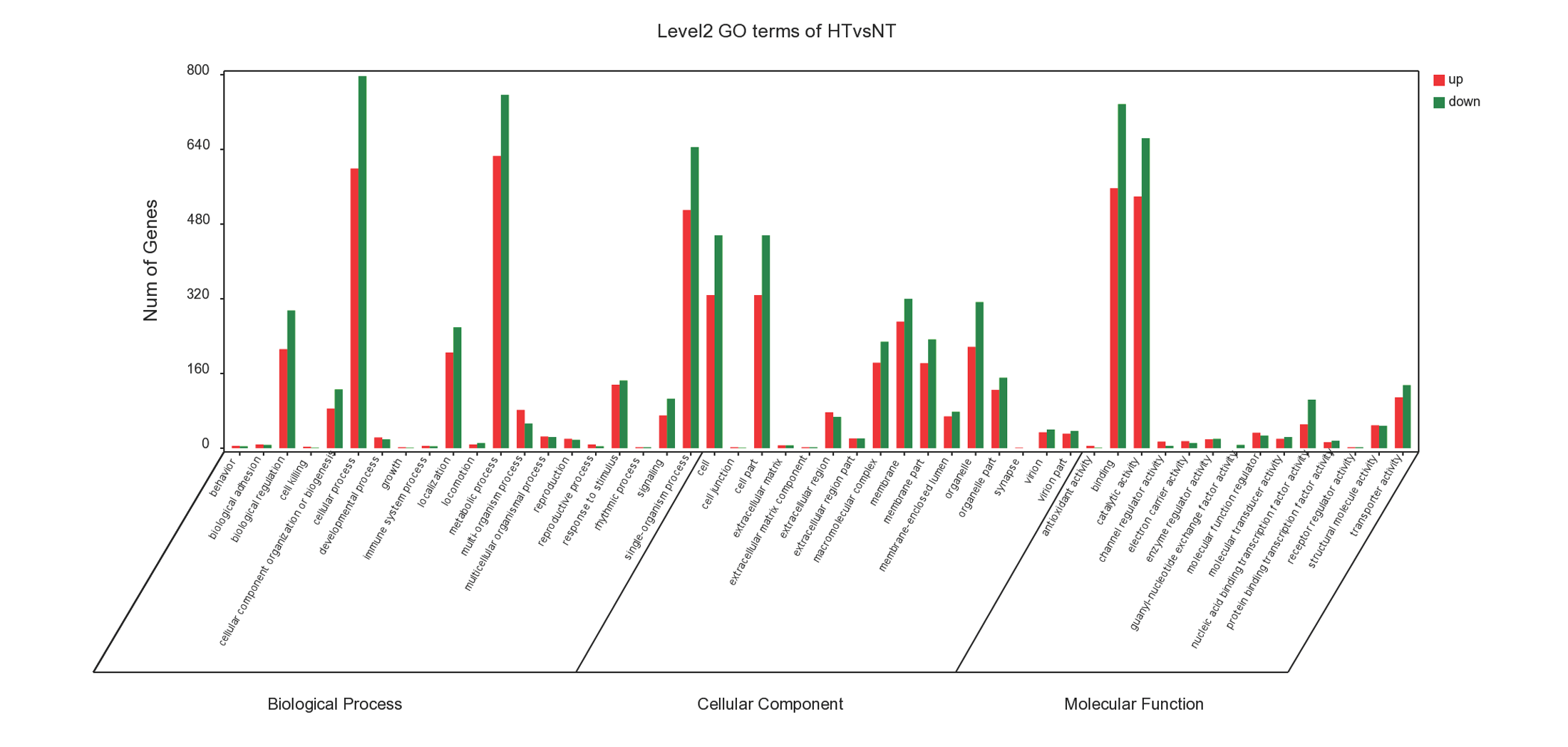

Supplement: S3 Fig — (TIF) [file pone.0186040.s003.tif]

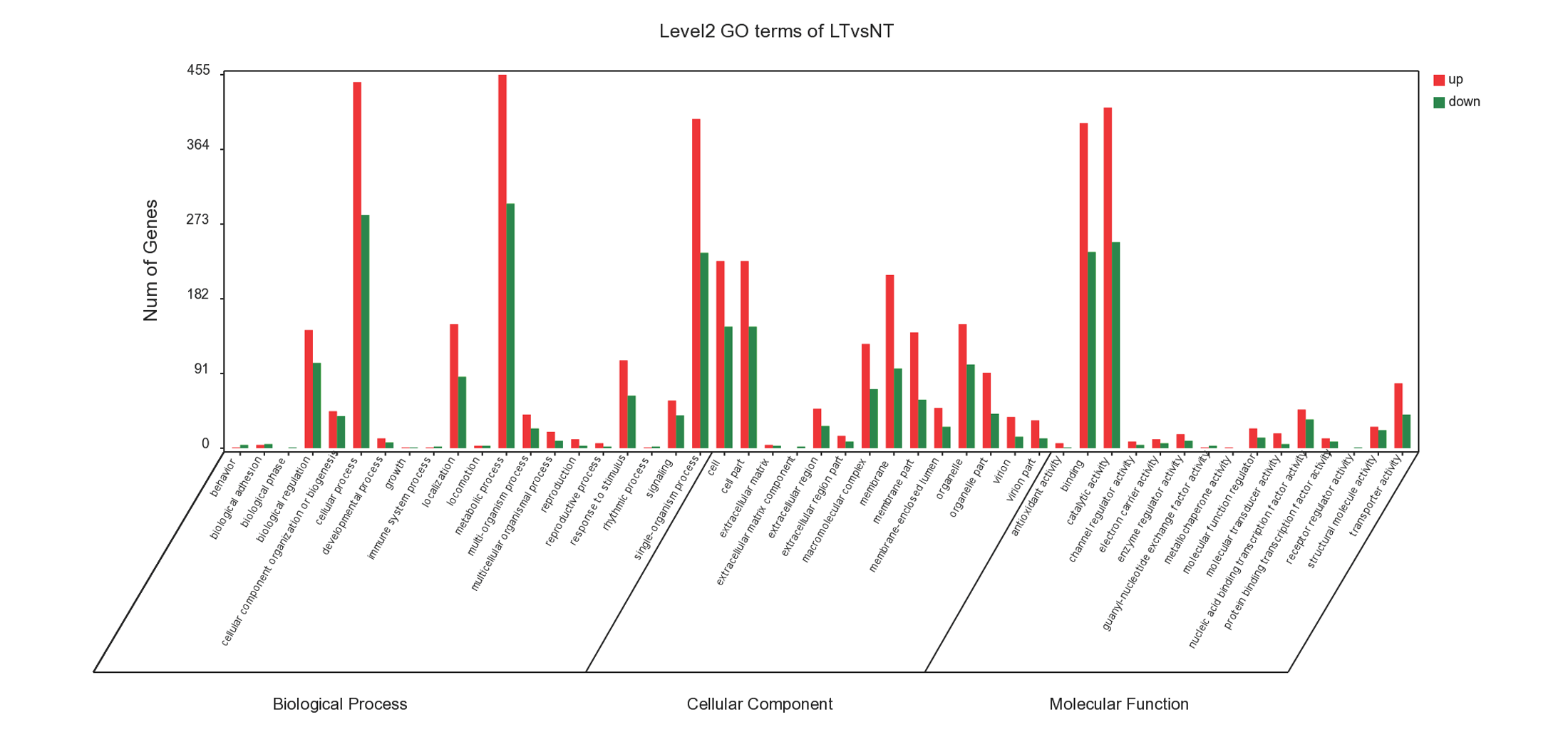

Supplement: S4 Fig — (TIF) [file pone.0186040.s004.tif]

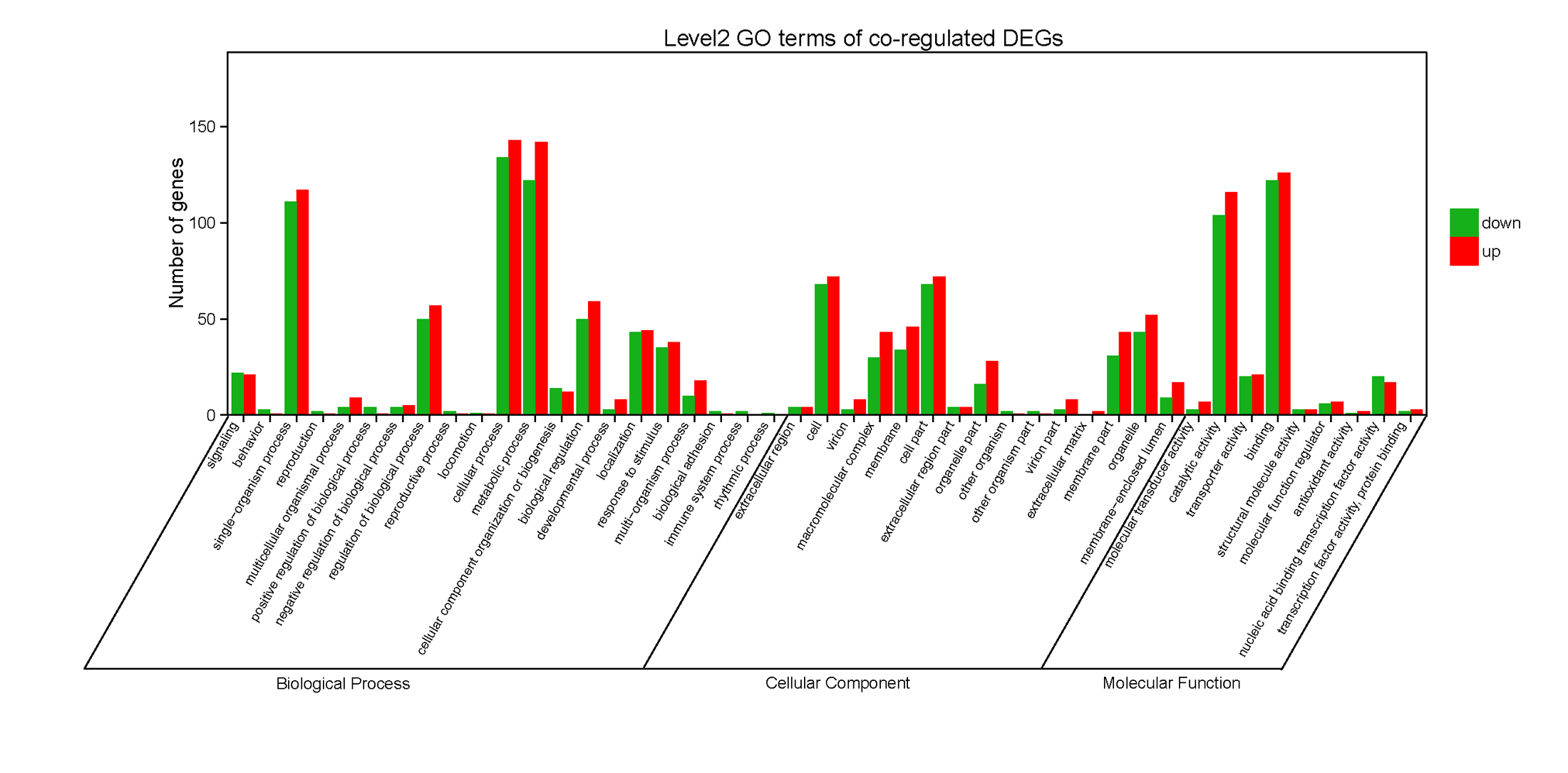

Supplement: S5 Fig — (TIF) [file pone.0186040.s005.tif]
